# Diagnostic and Prognostic Value of Three microRNAs in Environmental Asbestiform Fibers-Associated Malignant Mesothelioma

**DOI:** 10.3390/jpm11111205

**Published:** 2021-11-15

**Authors:** Veronica Filetti, Carla Loreto, Luca Falzone, Claudia Lombardo, Emanuele Cannizzaro, Sergio Castorina, Caterina Ledda, Venerando Rapisarda

**Affiliations:** 1 Human Anatomy and Histology, Department of Biomedical and Biotechnology Sciences, University of Catania, 95123 Catania, Italy; verofiletti@gmail.com (V.F.); carla.loreto@unict.it (C.L.); 2 Epidemiology Unit, IRCCS Istituto Nazionale Tumori “Fondazione G. Pascale”, 80131 Naples, Italy; l.falzone@istitutotumori.na.it; 3 Human Anatomy, Department of Medical and Surgical Sciences and Advanced Technologies, University of Catania, 95123 Catania, Italy; claudia.lombardo@unict.it (C.L.); sergio.castorina@unict.it (S.C.); 4 Occupational Medicine, Department of Sciences for Health Promotion and Mother and Child Care, University of Palermo, 90128 Palermo, Italy; emanuele.cannizzaro@unipa.it; 5 Occupational Medicine, Department of Clinical and Experimental Medicine, University of Catania, Via Santa Sofia 87, 95123 Catania, Italy; vrapisarda@unict.it

**Keywords:** fluoro-edenite, asbestos, malignant mesothelioma, microRNA, diagnosis, prognosis

## Abstract

Fluoro-edenite (FE) is an asbestiform fiber identified in Biancavilla (Sicily, Italy). Environmental exposure to FE has been associated with a higher incidence of malignant mesothelioma (MM). The present study aimed to validate the predicted diagnostic significance of hsa-miR-323a-3p, hsa-miR-101-3p, and hsa-miR-20b-5p on a subset of MM patients exposed to FE and matched with healthy controls. For this purpose, MM tissues vs. nonmalignant pleura tissues were analyzed through droplet digital PCR (ddPCR) to evaluate differences in the expression levels of the selected miRNAs and their MM diagnostic potential. In addition, further computational analysis has been performed to establish the correlation of these miRNAs with the available online asbestos exposure data and clinic-pathological parameters to verify the potential role of these miRNAs as prognostic tools. ddPCR results showed that the three analyzed miRNAs were significantly down-regulated in MM cases vs. controls. Receiver operating characteristic (ROC) analysis revealed high specificity and sensitivity rates for both hsa-miR-323a-3p and hsa-miR-20b-5p, which thus acquire a diagnostic value for MM. In silico results showed a potential prognostic role of hsa-miR-101-3p due to a significant association of its higher expression and increased overall survival (OS) of MM patients.

## 1. Introduction

The types of minerals forming fibers that have been used commercially and that are known by the term “asbestos” include serpentine (chrysotile) and fibrous amphiboles cummingtonite-grunerite (amosite asbestos), actinolite, anthophyllite, riebeckite (crocidolite asbestos), anthracite, and tremolite [1,2]. Such fibers represent an environmental health problem as chronic exposure to these minerals has been associated with respiratory diseases, including cancer [3,4].

Additionally, exposure to several other types of mineral particles found in the natural environment and termed “naturally occurring asbestos” (NOA) such as fibers of the minerals erionite, winchite, magnesio-riebeckite, Libby asbestos, richterite, antigorite, and fluoro-edenite (FE) have also been associated with malignant mesothelioma (MM) [1,2].

FE is a calcium amphibole of transparent and intense yellow color [5]. This silicate has several properties similar to the asbestos group [6,7]; in particular, it presents the same morphological and compositional aspect of the two fibrous phases tremolite and actinolite [2]. However, the peculiar feature of Biancavilla FE is its composition, characterized by high aluminum, fluorine, and sodium contents, compared with other known oncogenic minerals [8].

The International Agency of Research on Cancer (IARC) classified the FE fibers as carcinogenic to humans [9]. FE fibers effects similar to those already reported after exposure to asbestos fibers have been shown by several epidemiological studies [10,11,12,13,14,15,16,17]. Several studies have reported an increased incidence of malignant neoplasms affecting the pleura, the peritoneum, and the lung, after chronic inhalation of FE fibers [18,19,20]. All data suggest that FE fibers exposure is caused by environmental contamination and it is not due to specific occupational tasks [18]. The environmental exposure estimate is responsible for 10.8% of MM cases in Italy [21]. Material from the quarry of Monte Calvario (Biancavilla, Italy) has been used for about 50 years for local construction [10,12,22], and none of the residents diagnosed for MM had been significantly exposed to asbestos during their occupational activities [23].

Local inflammation in the lung and pleura is among the most recognized toxic effects of FE fibers exposure predisposing the individuals to MM development [24,25]. At present, MM is still considered a lethal cancer characterized by a considerable period of latency (≥30–60 years) [26] and late diagnosis that determines bad prognosis and quality of life and unresponsiveness to presently available treatments [27]. To date, there are no diagnostic tools with high sensitivity and specificity that can be used to perform an early diagnosis of MM in asymptomatic people. Many biomarkers have been proposed for the screening and diagnosis of MM in exposed subjects [28,29,30,31,32]. The unique Food and Drug Administration (FDA)-approved biomarker for MM is mesothelin [33,34,35], but its poor sensitivity limits the diagnosis of MM [36]. In this context, several studies demonstrated how miRNAs may be used as effective biomarkers of environmental contamination and exposure to toxic substances [37,38]. Furthermore, recent studies demonstrated that microRNAs (miRNAs) play an important role in MM biology and they have the potential to be considered as good non-invasive diagnostic and prognostic biomarkers and therapeutic targets for cancer [39,40,41]. In particular, Micolucci and colleagues [42] propose an early diagnosis of MM or a tool to monitor the therapy sensitivity through the expression levels analysis of several MM-associated miRNAs designated as “mesomiRs” [42,43].

This study aimed to evaluate the expression levels of a set of miRNAs previously identified and already validated in MM cellular models [44] to confirm their potential use as diagnostic biomarkers for MM. In this study, for the first time, hsa-miR-323a-3p, hsa-miR-101-3p, and hsa-miR-20b-5p have been analyzed in FE-mediated MM tissues vs. nonmalignant pleura tissues. Furthermore, an in silico analysis has been performed to evaluate the prognostic and therapeutic role of these three miRNAs. With this research work, we would contribute to basic biomarkers research, and we hope to transfer these results to clinical practice.

## 2. Materials and Methods

### 2.1. Patients and Samples Collection

Tissue specimens of ten cases of malignant mesothelioma and eight cases of healthy pleural mesothelium were retrospectively analyzed. Formalin-fixed and paraffin-embedded (FFPE) tissue specimens were obtained from the biobank of the Section of Anatomic Pathology, Department Gian Filippo Ingrassia, University of Catania. The exclusion criteria adopted in the choice of the cases were the following: (i) it was not possible to obtain additional slides from FFPE blocks for the analysis; (ii) no representative neoplastic tissue was contained in FFPE blocks. No written informed consent was necessary because of the retrospective nature of the study; the study protocols conformed to the ethical regulations of the Helsinki Declaration.

The cohort of patients of Biancavilla with FE-mediated MM was composed of six men and four women (mean age: 68.4 ± 13.9 years; age range: 50–93 years). Agreeing to the World Health Organization (WHO) criteria, six cases were histologically classified as epithelioid, three were classified as biphasic subtypes, and one was classified as sarcomatoid [21]. The cohort of control cases was composed of eight men (mean age: 44 ± 25.5 years; age range: 15–76 years). These patients did not live in Biancavilla, and they did not show oncological pathologies but pulmonary emphysema (*n* = 3) and pleurisy (*n* = 5). Data including MM cases and controls are summarized in Table 1.

Freshly cut sections of FFPE tissue, each with a thickness of 20 µm, were obtained using a rotary microtome. Two sections for each sample were collected and stored at room temperature.

### 2.2. RNA Isolation and RT

Total RNA containing small non-coding RNA was extracted from FFPE tissue using a miRNeasy FFPE Kit (QIAGEN; Hilden, Germany) according to the manufacturer’s recommended protocol (miRNeasy FFPE Handbook 01/2020). The RNA extraction and quantification were performed as previously described [44]. All samples were then stored at −80 °C until use. The purified RNA was reverse transcribed into cDNA as previously described [44].

### 2.3. Droplet Digital PCR

As previously described, a customized droplet digital PCR (ddPCR) assay was used to amplify hsa-miR-323a-3p, hsa-miR-101-3p, and hsa-miR-20b-5p [45]. The reaction mixture was made by combining ddPCR Supermix for probes (no dUTP) (cat. n. 1,863,010—Bio-Rad Laboratories), TaqMan Advanced miRNA Assays specific for each miRNA (cat. no. 477863, 477804, 477,853—Thermo Fisher Scientific), a miR-Amp cDNA sample and PCR water. The cartridge was loaded with 20 microliters of PCR reaction and 70 μL of Droplet Generation Oil (cat. no. 1,863,005—Bio-Rad Laboratories) in appropriate wells, and then Droplet Generator QX200 was used to generate droplets. Subsequently, the generated droplets were amplified using a C 1000 Touch Thermal Cycler (Bio-Rad Laboratories) at the cycling conditions previously described [44]. A non-template control (NTC) was inserted for each probe.

After the amplification, the droplets were read using a QX200 Droplet Reader (Bio-Rad Laboratories). Finally, the absolute quantification of targets was performed using QuantaSoft software, version 1.7.4 (QuantaSoft, Prague, Czech Republic), as previously described [44,46].

### 2.4. In Silico Analysis

To clarify the role of hsa-miR-323a-3p, hsa-miR-101-3p, and hsa-miR-20b-5p in MM, different computational tools were used.

By consulting the Catalogue of Somatic Mutation In Cancer (COSMIC) (http://cancer.sanger.ac.uk/cosmic (accessed on 20 September 2021) it was possible to identify the 20 most mutated genes that are known to be involved in MM development and therefore have a dysregulated expression. The selection was performed using the search term “Malignant Mesothelioma” including the terms “Pleura” in tissue selection and “Mesothelioma” in histology selection.

Subsequently, using the bioinformatics prediction tool microRNA Data Integration Portal (mirDIP version 4.1.11.2, Database version 4.1.0.3, November 2020) (http://ophid.utoronto.ca/mirDIP, accessed on 20 September 2021) [47,48], the interaction levels between the miRNA previously identified via computational analysis and the main genes mutated and altered in MM were evaluated.

Furthermore, the clinical implication of the three analyzed miRNAs was assessed through the clinic-pathological data and the miRNA expression profiles analysis contained in The Cancer Genome Atlas Mesothelioma (TCGA-MESO) database and downloaded using the online exploration tool UCSC Xena Browser (https://xenabrowser.net/, accessed on 21 September 2021) [49]. In particular, the TCGA database was used to verify if the three miRNAs here analyzed were dysregulated in MM according to asbestos exposure, tumor stage, and patient survival. A total of 17 MM patient-related datasets were found containing a total of 87 MM samples (35 exposed to asbestos, 49 not exposed to asbestos, 3 excluded due to lack of useful information). The datasets contained the expression levels of 1,964 different miRNAs, but we focused on hsa-miR-323a-3p, hsa-miR-101-3p, and hsa-miR-20b-5p for further investigation.

### 2.5. Statistical Analysis

The Shapiro‒Wilk normality test was applied for the calculation of the distribution of hsa-miR-323a-3p, hsa-miR-101-3p, and hsa-miR-20b-5p expression levels observed with ddPCR and deposited on the TCGA-MESO database. The Mann‒Whitney test was utilized for the comparison between miRNAs expression of MM samples and healthy controls. Receiver operating characteristic (ROC) curves were obtained to evaluate the specificity and sensitivity of the analyzed miRNAs. An unpaired Student t-test and one-way ANOVA test were used for assessing the statistical differences existing between the expression levels of hsa-miR-323a-3p, hsa-miR-101-3p, and hsa-miR-20b-5p reported in the TCGA-MESO database according to the asbestos exposure and the MM tumor stages, respectively. Cancer-specific survival analysis was performed using the Kaplan‒Meier method, and for comparison of the survival curves, the Mantel-Cox log-rank test was used.

A value of *p* < 0.05 was considered statistically significant. The graphs were plotted using Prism for Windows version 7.00 (Graphpad Software; San Diego, CA, USA), and data were represented as the mean with SD.

## 3. Results

### 3.1. Evaluation of miRNA Expression Profiling Using ddPCR

The expression of hsa-miR-323a-3p, hsa-miR-101-3p, and hsa-miR-20b-5p was examined in MM tissues and relative controls of healthy pleural mesothelium.

The Shapiro‒Wilk normality test showed that the expression levels of the three miRNAs analyzed in MM cases and healthy controls differed significantly from a normal distribution.

**Figure 1:**
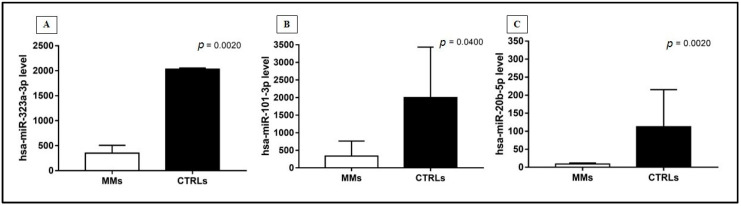
MiRNAs expression, reported as number of copies/uL of reaction, in MM cases and healthy controls, through the Mann‒Whitney test, according to: (**A**) hsa-miR-323a-3p, (**B**) hsa-miR-101-3p, (**C**) hsa-miR-20b-5p. Data were represented as the mean with SD.

The comparison between tumor and normal tissues showed a different expression of the three miRNAs analyzed in MM and healthy controls. The expression levels of hsa-miR-323a-3p, hsa-miR-101-3p, and hsa-miR-20b-5p in MM cases were significantly lower compared to controls (Figure 1; Supplementary Figure S1). There was a statistically significant trend of down-regulation observed for the three selected miRNAs analyzed in MM cases vs. controls.

**Figure 2:**
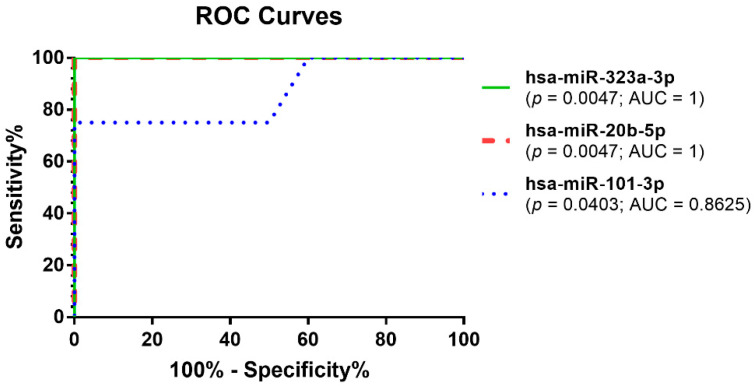
ROC curves demonstrated the diagnostic value of hsa-miR-323a-3p (100% sensitivity and 100% specificity), hsa-miR-20b-5p (100% sensitivity and 100% specificity), and hsa-miR-101-3p (100% sensitivity and 40% specificity).

To evaluate the sensitivity and specificity of these miRNAs and their role as novel, promising diagnostic biomarkers for MM, ROC (receiver operating characteristic) curves were calculated (Figure 2). ROC analysis revealed high specificity and sensitivity rates for both hsa-miR-323a-3p and hsa-miR-20b-5p. In particular, the sensitivity and specificity for hsa-miR-323a-3p and hsa-miR-20b-5p were 100% and 100% (AUC (area under the curve) = 1). For hsa-miR-101-3p the sensitivity was 100% and the specificity was 40% (AUC = 0.8625). However, the AUCs of all analyzed miRNAs were statistically significant (*p* < 0.05).

### 3.2. In Silico Interaction between miRNAs and Main Genes Involved in Malignant Mesothelioma

Gene target analysis performed using the bioinformatics tool mirDIP showed the level of interaction of the three computationally identified miRNAs with the main altered or mutated gene in MM.

**Figure 3:**
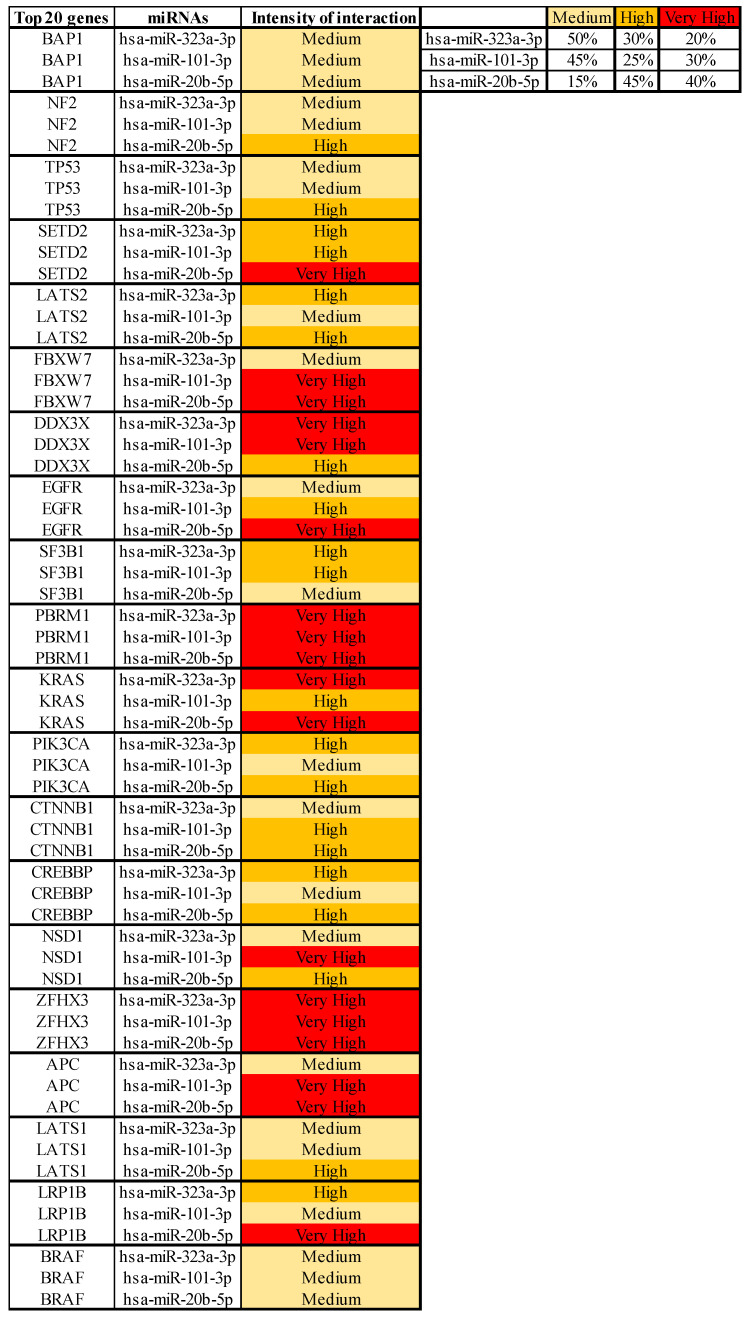
Interaction between selected miRNAs and main altered genes in MM via mirDIP gene target analysis. For each miRNA the level of interaction with the 20 genes involved in MM is reported. The intensity of interaction is highlighted with a color scale ranging from yellow (medium interaction) to red (very high interaction).

We took into account 20 different genes obtained from COSMIC and the mirDIP analysis revealed that the three evaluated miRNAs were able to interact with all genes involved in MM with high levels of intensity. These genes were: BAP1 (24%), NF2 (18%), TP53 (12%), SETD2 (7%), LATS2 (5%), FBXW7 (2%), DDX3X (3%), EGFR (1%), SF3B1 (2%), PBRM1 (2%), KRAS (1%), PIK3CA (1%), CTNNB1 (1%), CREBBP (2%), NSD1 (2%), ZFHX3 (2%), APC (1%), LATS1 (2%), LRP1B (2%), BRAF (1%). In particular, for each miRNA the intensity of interaction was reported to be from medium to very high specificity. All miRNAs showed very high and high interaction levels with at least 50% of the genes analyzed, suggesting a potential role of these miRNAs in the development of MM. According to this analysis, hsa-miR-20b-5p showed very high and high interaction levels with 85% of the genes mutated in MM. The genes linked with higher levels of intensity by miRNAs were found to be PBRM1 and ZFHX3, which showed in all cases very high levels of interaction. In addition, the KRAS gene showed very high levels of interaction with hsa-miR-323a-3p and hsa-miR-20b-5p and high levels of interaction with hsa-miR-101-3p. In opposition, the genes associated with medium levels of intensity for all miRNAs were found to be BAP1 and BRAF. None of the genes analyzed showed low levels of interaction intensity with the miRNAs evaluated (Figure 3).

According to this analysis, these three miRNAs can target and modulate both tumor suppressor and oncogene genes playing a potentially key role in tumor cell development.

### 3.3. In Silico Interaction between miRNAs and Asbestos Exposure, Tumor Stage, and Patient Survival

The Shapiro‒Wilk normality test showed that the expression levels of the three miRNAs contained in the TCGA-MESO database have a normal distribution.

**Figure 4:**
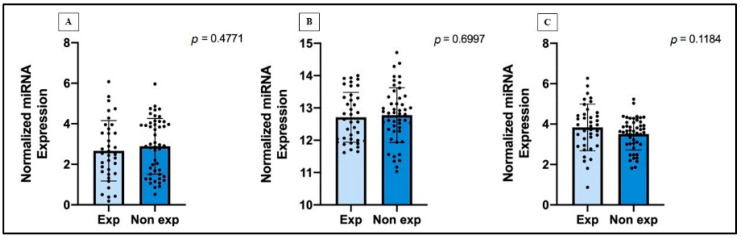
Normalized miRNAs expression in MM cases exposed and non-exposed to asbestos, through unpaired Student t-test, according to: (**A**) hsa-miR-323a-3p, (**B**) hsa-miR-101-3p, (**C**) hsa-miR-20b-5p. Data are represented as the mean with SD.

The analysis of miRNAs expression levels according to the asbestos exposure data contained in the TCGA-MESO database revealed that the expression levels of hsa-miR-323a-3p, hsa-miR-101-3p, and hsa-miR-20b-5p did not change significantly in MM patients exposed and not exposed to asbestos fibers (Figure 4).

**Figure 5:**
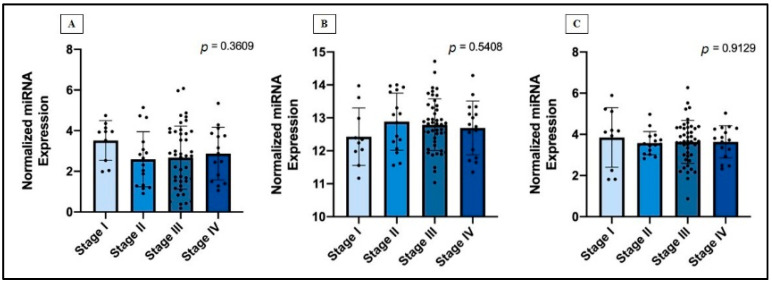
Normalized miRNAs expression in different MM stages, through one-way ANOVA test, according to: (**A**) hsa-miR-323a-3p, (**B**) hsa-miR-101-3p, (**C**) hsa-miR-20b-5p. Data are represented as the mean with SD.

The analysis of miRNAs expression levels according to the clinic-pathological data contained in the TCGA-MESO database showed that the expression levels of hsa-miR-323a-3p, hsa-miR-101-3p, and hsa-miR-20b-5p did not change significantly in MM patients with different tumor stages (Figure 5).

**Figure 6:**
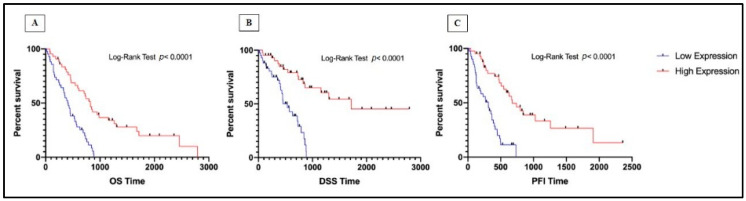
Kaplan‒Meier survival curve of hsa-miR-101-3p expression in MM patients according to: (**A**) OS time; (**B**) DSS time; (**C**) PFI time.

Finally, considering the median overall survival (OS), the disease-specific survival (DSS), and the progression-free interval (PFI) between high and low miRNAs expression, significance for hsa-miR-101-3p (*p* < 0.0001) was shown. In particular, there was an association of high hsa-miR-101-3p expression and increased OS time (Figure 6A), DSS time (Figure 6B), and PFI time (Figure 6C). On the contrary, hsa-miR-323a-3p and hsa-miR-20b-5p did not show significant results in MM patients’ survival.

## 4. Discussion

In this study, for the first time, hsa-miR-323a-3p, hsa-miR-101-3p, and hsa-miR-20b-5p have been analyzed in MM tissues vs. nonmalignant pleura tissues. All tested miRNAs in MM tissue showed a down-regulation compared to controls.

The translational data obtained for hsa-miR-101-3p in the present study were totally in accordance with our previous computational study which showed a significant down-regulation in MM samples compared to controls [44]. Furthermore, this miRNA showed a prognostic value for MM because the in silico analyses performed here demonstrated a significant association between hsa-miR-101-3p high expression levels and increased OS. In line with our research, a study by Ramírez-Salazar et al. [50] demonstrated that the targets of the down-regulated hsa-miR-101-3p in MM were significantly enriched in pathways in cancer, including the signaling molecule mitogen-activated protein kinase 1 (MAPK1), the transcription factor v-ets erythroblastosis virus E26 oncogene homolog 1 (ETS1), and the mesenchymal transition-associated molecule frizzled class receptor 4 (FZD4). Multiple down-regulated miRNAs targeted multiple common oncogenic genes, as their reduced expression could increase the expression of these genes and consequently promote tumorigenesis [50]. This explains why an increase in hsa-miR-101-3p levels increases the survival of MM patients, in which the high expression of hsa-miR-101-3p decreases the expression of these oncogenic genes and consequently counteracts tumorigenesis. Interestingly, although hsa-miR-20b-5p showed higher interaction levels with the most altered genes in MM, the expression levels of this miRNA were not associated with the overall survival or progression-free survival of patients. This phenomenon may be related to the multiple binding activities of miRNAs exerted towards different genes. Our results may support the hypothesis that miRNAs reach their biological impact by targeting multiple genes with similar biological effects. Therefore, although hsa-miR-20b-5p showed high levels of interaction with the genes analyzed, hsa-miR-101-3p can activate a more complex network of genes involved in the progression of tumors and the survival of patients, as demonstrated in other studies [50,51,52,53].

Of note, all the miRNAs previously identified and here validated in human samples have already been associated with the development of different tumors, including breast cancer, glioblastoma multiform, lymphomas, etc. [54,55,56].

It is noteworthy that the results obtained for hsa-miR-323a-3p and hsa-miR-20b-5p were opposite to those obtained in our previous in silico and in vitro analysis, however, for these miRNAs ROC analysis revealed high sensitivity and specificity in correctly distinguishing MM and normal samples.

The computational analysis performed to further establish the functional role of these three miRNAs in MM pathogenesis has shown that these miRNAs can target and modulate both tumor suppressor and oncogene genes playing a potentially key role in tumor cell development. In particular, the genes targeted with higher levels of interaction by the selected miRNAs were PBRM1, ZFHX3, and KRAS. The alteration of the expression levels of PBRM1 was associated with the development of both renal cell carcinoma and MM [57]. Recent evidence suggests that PBRM1 genomic alterations are strongly associated with neoantigen production and responsiveness to immune checkpoint inhibitors (ICIs), therefore, the analysis of the expression levels and gene mutation affecting this gene may be predictive for the therapeutic choice in MM. ZFHX3, named also *ATBF1*, has been widely associated with the development of lung cancer when dysregulated. In addition, the analysis of ZFHX3 is also a positive prognostic biomarker for patients treated with ICIs [58]. Similarly, other studies have demonstrated the predictive role of *KRAS* alteration for the treatment of lung cancer [59]. In addition, a recent study also established an important role of KRAS mutation in the development of MM [60].

Several studies have tried to identify novel diagnostic biomarkers for the management of MM patients, however, the currently available diagnostic strategies, mainly based on the evaluation of tumor biomarkers such as calretinin, cytokeratin 5, podoplanin, mesothelin, osteopontin, hyaluronic acid, fibulin-3 [28], vascular endothelium growth factor [30], aquaporin-1 [29], high mobility group box 1 [31], and macroH2A.1 [32], often fail to correctly diagnose MM due to the low rates of sensitivity and specificity of these biomarkers. 

To date, liquid biopsy is emerging as a helpful tool for non-invasive diagnosis, screening, prognosis, and stratification of cancer patients [61,62,63] and to characterize tumor heterogeneity [64]. The literature already proposes an early diagnosis of MM through the expression levels analysis of several “mesomiRs” [42]. Circulating miRNA-126-3p, miRNA-625-3p, and miRNA-103a-3p in blood paired with with mesothelin and fibulin-3 have been suggested as potential diagnostic biomarkers of MM [42]. This approach could avoid the histopathological and immunohistochemistry techniques used as the standard for the late diagnosis of pleural biopsies [21]. It could be particularly helpful to study and subsequently use a combination of several proteins and molecular markers to improve diagnostic accuracy.

For this purpose, droplet digital PCR (ddPCR) investigations as well as in silico analysis were performed to assess the functional role of the selected miRNAs and their predictive value for MM patients’ diagnosis and prognosis.

Our results indicated that by increasing the number of samples these miRNAs should be further evaluated not just as diagnostic tools, but additionally as prognostic predictors. Thus, their potential as therapeutic targets could be explored by assessing their molecular role. Another key task will be the validation of their targets and regulators, which would clarify how miRNAs induce or suppress critical pathways involved in the carcinogenesis triggered by carcinogenic fibers exposure.

## 5. Conclusions

Our goal is the validation of these results in a subset of patients chronically exposed to FE using liquid biopsy, to provide a minimally invasive screening tool for the secondary prevention of MM. Early detection of circulating tumor biomarkers and tumor DNA represents one of the most promising strategies to enhance the survival of cancer patients by increasing treatment efficiency [63,64]. Besides these preliminary data, further studies will be designed for the validation of “mesomiRs” with diagnostic potential, alone or in combination with other protein biomarkers, to test their clinical role in high-risk individuals. Certainly, a limitation of the work is the low number of samples available and the reliance on the diagnostic values (sensitivity and specificity) of miRNAs based on the obtained ROC curves until the evaluation on the independent test sample. It would also be interesting to have available a cohort of subjects exposed to FE fibers but in the absence of tumors. This comparison could highlight any similarities or epigenetic differences not only between oncological and non-oncological subjects but also between those exposed to carcinogenic fibers and those not. Moreover, further basic research studies should be aimed at investigating the molecular pathways that are regulated by aberrantly expressed miRNAs.

## Figures and Tables

**Table 1:** Features of the fluoro-edenite (FE)-related malignant mesothelioma (MM) cases and controls.

	Age Range (Years)	Mean Age (Years)	Gender	Pathologies	Pathological Subtype	Survival Time Range (Days)	Mean Survival Time (Days)
**Cases (*n* = 10)**	50–93	68.4 ± 13.9	60% men, 40% women	100% malignant mesothelioma	60% epithelioid, 10% sarcomatoid, 30% biphasic	45–1800	579 ± 525
**Controls (*n* = 8)**	15–76	44 ± 25.5	100% men	37.5% pulmonary emphysema, 62.5% pleurisy			

## Supplementary Materials

The following are available online at https://www.mdpi.com/article/10.3390/jpm11111205/s1, Figure S1: ddPCR amplification signals obtained for NTC sample, MM sample and control sample for the three investigated miRNAs.
