# Supplementary material for: Diagnostic and Prognostic Value of Three microRNAs in Environmental Asbestiform Fibers-Associated Malignant Mesothelioma
Source: J Pers Med. 2021 Nov 15;11(11):1205. doi: 10.3390/jpm11111205 (PMC8618926; doi:10.3390/jpm11111205)
Supplement: Supplementary file 1 [file jpm-11-01205-s001.zip › jpm-1411906-supplementary.pdf]

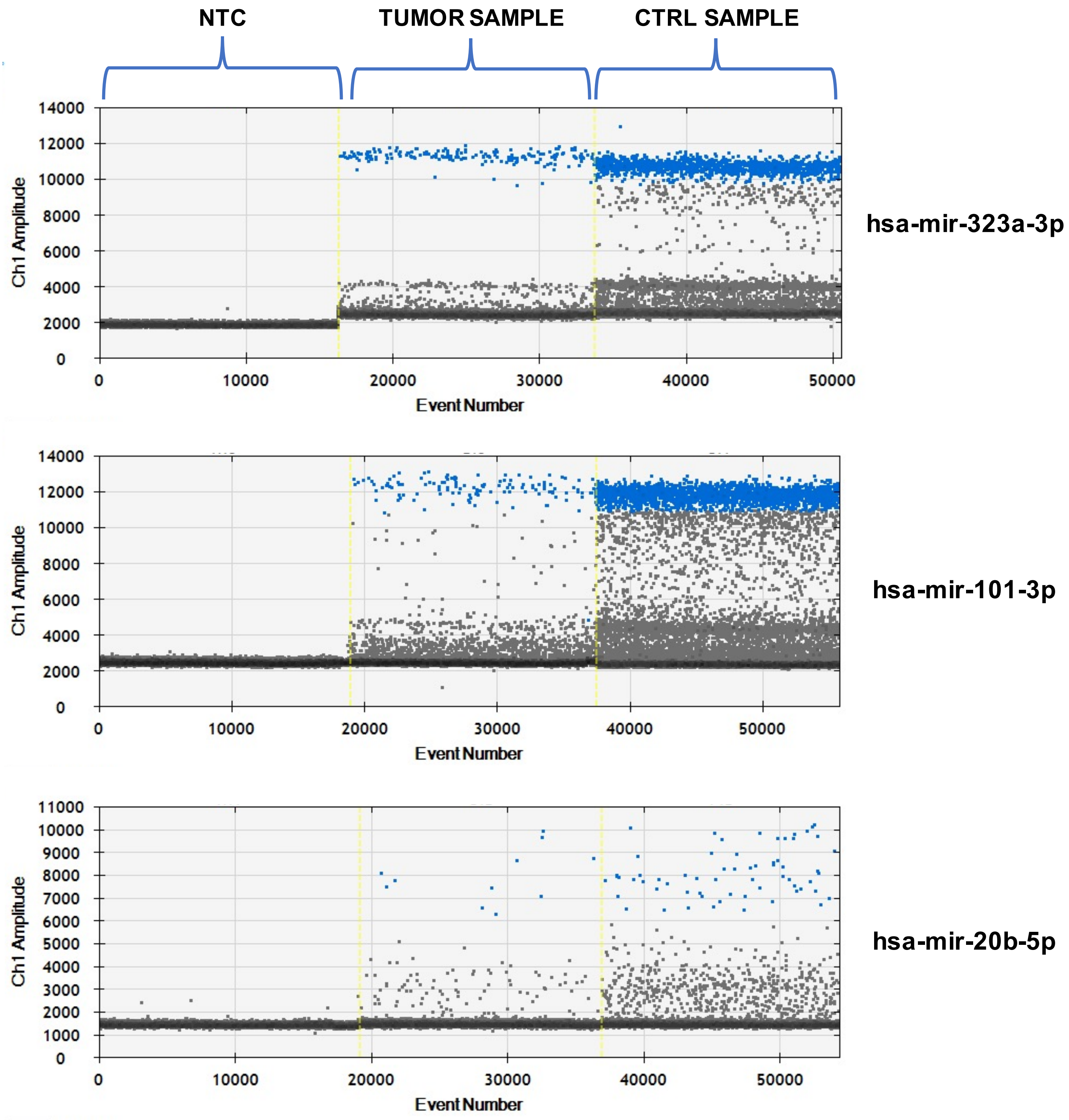

Figure S1: ddPCR amplification signals obtained for NTC sample, MM sample and control sample for the three investigated miRNAs.
